# Functional alignment in total knee arthroplasty is an umbrella term—A call for better definition and reporting quality!

**DOI:** 10.1002/ksa.12774

**Published:** 2025-07-13

**Authors:** Antonio Klasan, Dragan Jeremic, Thomas Neri, Thomas Jan Heyse, Michael T. Hirschmann

**Affiliations:** ^1^ AUVA UKH Steiermark Graz Austria; ^2^ Johannes Kepler University Linz Austria; ^3^ St. Vinzenz Hospital Brakel Germany; ^4^ Department of Orthopaedic Surgery University Hospital Centre of Lyon France; ^5^ Inter‐University Laboratory of Human Movement Science University Lyon ‐ University Jean‐Monnet Saint‐Étienne Saint‐Étienne France; ^6^ Red Cross Hospital Frankfurt Germany; ^7^ Department of Orthopedic Surgery and Traumatology Kantonsspital Baselland Bruderholz Switzerland; ^8^ Department of Clinical Research, Research Group Michael T. Hirschmann, Regenerative Medicine & Biomechanics University of Basel Basel Switzerland

**Keywords:** functional alignment, kinematic alignment, robotics, total knee arthroplasty

## Abstract

**Purpose:**

Functional alignment (FA) aims to achieve a symmetric, rectangular extension gap and a rectangular or trapezoidal flexion gap, positioning the components in a manner that reduces the compromises to the soft tissue envelope. Because the FA surgeon can accomplish this goal with a multitude of adjustments to the components' position or soft tissue balance, the purpose of the present study was to analyze the possible paths for achieving FA.

**Methods:**

Ten knees undergoing robotic total knee arthroplasty (TKA) were analyzed. Based on the intraoperatively acquired data, a macro was created to perform a post hoc analysis of the 10 possible paths to align a TKA functionally: (1) starting from mechanical alignment (MA) or kinematic alignment (KA), (2) preserving either the position of the femoral or tibial component and (3) with equal, 1 mm gaps or with increased 90° lateral flexion laxity.

**Results:**

Ten different knee phenotypes were analyzed (Var‐Neu‐Neu 2x; Neu‐Neu‐Val; Val‐Val‐Val; Neu‐Val‐Neu; Var‐Neu‐Var 3x; Var‐Var‐Var; Var‐Var‐Val). On average, 3.5 different paths resulted in FA for each TKA (range: 0–8). Two TKAs (Val‐Val‐Val; Var‐Var‐Val) could not be functionally aligned using any of the ten evaluated paths. One TKA could be functionally aligned using eight different paths.

**Conclusion:**

Functionally aligning a TKA can be achieved through multiple adjustments, resulting in various implant positions and soft tissue balances. To better understand the different combinations behind FA, a more detailed nomenclature is needed, including which initial alignment (MA or KA) was utilized, which component's position (femoral or tibial) was preserved, and whether a rectangular or trapezoidal flexion space was targeted.

**Level of Evidence:**

Level IV, therapeutic study.

**Abbreviations**
aMA: adjusted/restricted MA
CPAK: coronal plane alignment of the knee
FA: functional alignment
FKP: functional knee phenotype
iKA: inverse KA
KA: kinematic alignment
MA: mechanical alignment
NEU: neutral
RAS: robotic‐assisted surgery
rKA: restricted KA
TKA: total knee arthroplasty
unKA: unrestricted KA
VAL: valgus
VAR: varus

## INTRODUCTION

Total knee arthroplasty (TKA) has found itself in a renaissance due to several pivoting moments in the last few years. First, not all legs are straight, and not all knees have pathological alignment requiring correction [3, 13, 14]. Second, long‐term data from kinematic alignment (KA) philosophy studies have shown outcomes and survival rates at least as good as mechanical alignment (MA) [9, 17]. Third, the strong push towards robotic‐assisted surgery (RAS), as an upgrade from navigation [24], observed on the market has demonstrated increased precision, regardless of the alignment strategy or the system used [5, 7, 11]. It is now common knowledge that systematic MA has a considerable number of limitations [1].

This has allowed a change towards personalized alignment strategies, which should be performed in a safe manner [6] as a way to potentially improve outcomes [12]. Multiple strategies exist: unrestricted KA (unKA) [16], restricted KA (rKA) [20], inverse KA (iKA) [26], adjusted/restricted MA (aMA) [26] and functional alignment (FA) [19]. FA requires intraoperative enabling technology such as robots or smart navigation tools [19, 20, 26]. unKA, aMA and rKA can be done manually [16, 22]. All robotic systems allow an analysis of the soft tissue envelope and adjustments of the components' position to balance the knee joint. It has been shown that at least minor adjustments will almost always be performed when FA is targeted with a robotic system [28]. Alternatively, sensor‐guided technology can also be used to achieve FA [8].

FA aims to achieve symmetric extension and flexion gaps and positions the components in a manner that minimizes changes and compromises to the soft tissue envelope. The initial starting position is either MA or KA, followed by virtual adjustments to achieve that goal. iKA, a tibia preserving FA, follows the same FA principles; however, only by adapting the position of the femoral component [26].

According to the definition of FA, medial tightness in extension is generally corrected by a varus correction of the tibial component and lateral tightness with a valgus correction of the femoral component [19]. For some proponents of FA, or if a posteriorly stabilized implant is used, the targeted gaps are symmetrical in extension and flexion. However, increased lateral laxity in 90° of flexion has also been described as functionally aligned [21]. The acceptable boundaries for tibial and femoral implant positions also vary. For instance, for the tibial component, either 3° or 6° of varus is allowed, all under the term FA [19].

Depending on the starting position, MA or KA, the joint kinematics is greatly influenced, which also drives the way the components' position is adjusted to achieve FA. As both implants can be adjusted in their respective position, achieving the targeted gaps can be performed via the tibia, the femur, or both. In iKA, the tibia should remain in KA alignment. From a purely kinematic perspective, however, the knee axes are in the femur, and not the tibia, so, in theory, the femur should be preserved to maintain femur‐driven kinematic principles. A pertinent question remaining is ‘Which component, therefore, should stay as close as possible to its anatomical position when planning and performing FA?’.

Current FA nomenclature fails to label the starting alignment (MA or KA) and which component's alignment is prioritized to be preserved when balancing the TKA, and what the targeted gaps actually are.

Hence, the study's primary goal in patients undergoing RAS‐TKA was to determine possible combinations for achieving FA. The study utilized MA and KA starting positions with strategies to preserve the initial femoral or tibial component position and to generate symmetric or asymmetric flexion gaps.

## METHODS

Included were 10 consecutive patients undergoing RAS TKA using the MAKO System (Stryker), with the TKA 2.0 software (Stryker), performed by a single surgeon in January 2025 (A.K.). Preoperative standing anteroposterior X‐rays are routinely performed, and each patient was classified according to the functional phenotype classification [15]. The most distal and most posterior points were used as reference points for the resection, as per protocol [23]. These were controlled by the surgeon before the case in order to match the CT measurements. Prior to the beginning of the surgery, all cases were planned according to the principles of unKA [16], removing equal amount of bone, for CT‐referenced cuts, 7 mm, irrespective of the coronal or axial degree. Deviating from KA principles, the posterior tibial slope was set at 3°. The implant positions were then recorded. Then, the same was performed using MA principles. Both components were oriented perpendicular to the mechanical axis in the coronal plane. The femoral component was rotated neutral to the surgical transepicondylar axis. The cutting depth was 7 mm off the proud side, and the posterior tibial slope was set at 3°. The implant positions were then recorded.

**Figure 1:**
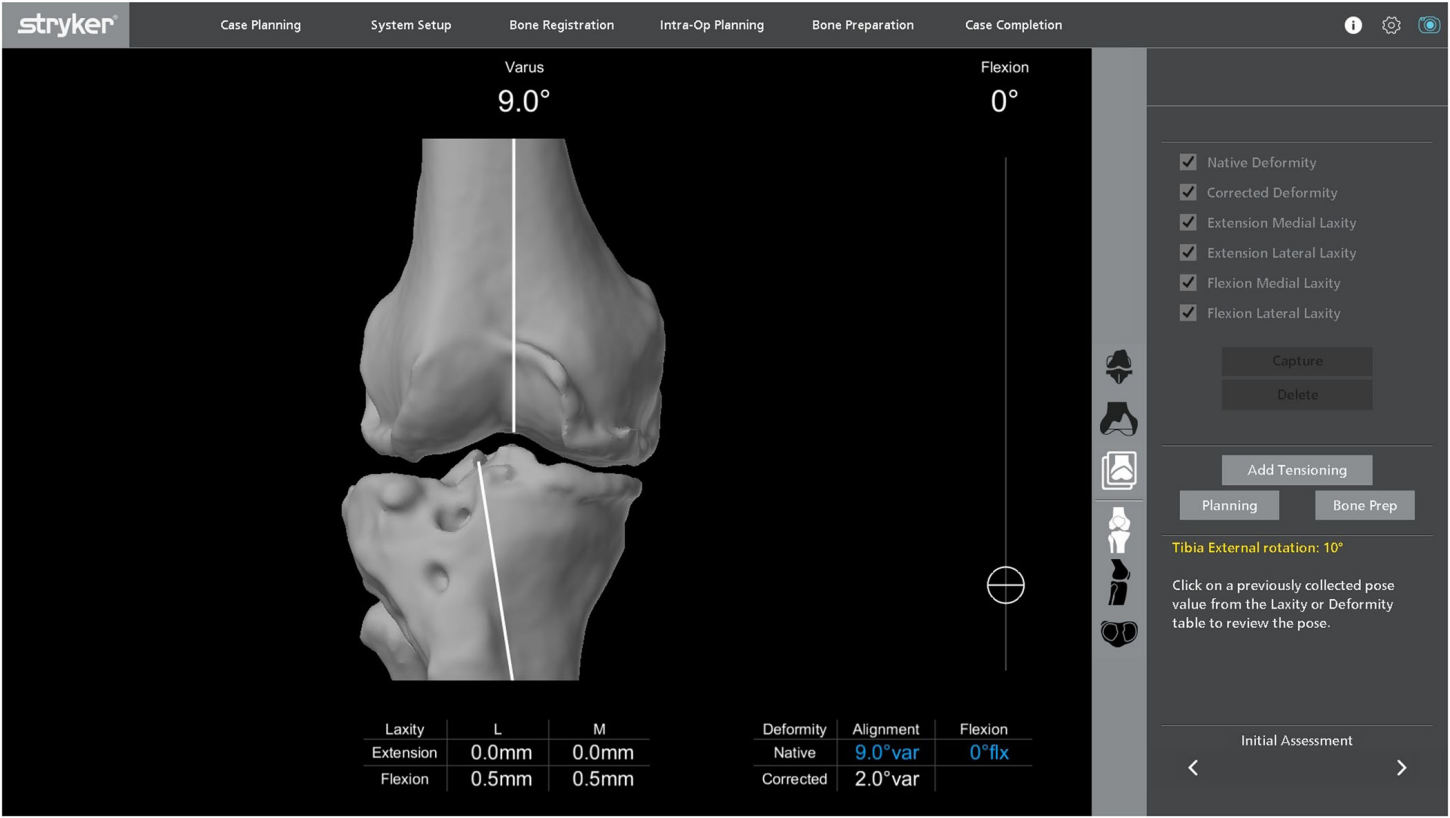
Intraoperative capturing of gaps and position using the proprietary Software (MAKO, Stryker).

The 2.0 software allows the surgeon to record all four gaps (i.e., medial and lateral gaps in extension and 90° flexion) individually; however, all four gaps are documented on one screen (Figure 1). Once registration was performed and the osteophytes removed, virtual gap balancing was performed. For the post‐hoc alignment analysis, a 1° correction equalled a 1 mm gap difference. A 1° tibial slope alteration resulted in a 0.5 mm gap change. If this was not observed in the case, the case was excluded.

### Post hoc alignment analysis

Once the case is completed, the MAKO software (Stryker) does not allow further changes of component alignment to alter the gaps. To compensate for this, using the initial alignment, resection depths and intraoperative gaps, a Microsoft Excel (Microsoft Corp) macro was created [28]. The 1° = 1 mm equation computed the gaps. Varus–valgus changes of the femoral component altered the extension gap; rotational changes altered the flexion gap. Varus–valgus changes of the tibial component altered both flexion and extension gaps, while the changes of posterior tibial slope altered only the flexion gap.

Both KA and MA were used as starting alignments for each case. Using both starting alignments, each case was then aligned by trying to preserve the initial femoral position (femur preserving) or trying to preserve the initial tibial alignment (tibia preserving). As per FA principles, symmetrical flexion and extension gaps were aimed for [19], or, as a secondary goal, a 90° lateral flexion laxity up to 6 mm greater than the medial gap was allowed [21]. If FA could not be achieved according to either of these strategies, both component positions were changed (hybrid). Ten possible combinations for achieving FA were evaluated and reported for each knee.

### Statistical analysis

An a priori power analysis as a superiority trial was performed. Using alpha of 0.05 and beta of 0.8, in order to detect a difference between 20% and 80% successful balancing, seven cases would be needed. Descriptive statistics were used to compare successful alignment strategies between the cases. Statistical analysis was performed using SPSS 29.0 (IBM).

## RESULTS

After applying the inclusion and exclusion criteria, four patients were excluded due to a MAKO software rounding error, rendering the 1 mm = 1° rule not applicable. There were 10 different functional knee phenotypes (FKPs) in the 10 included knees (Table 1). Neither the initial MA nor KA alignment resulted in FA in any of the 10 knees (Table 2).

**Table 1:** Knee phenotypes of included patients.

Patient number	Knee phenotype
1	VAR_HKA_9° NEU_FMA_0° NEU_TMA_0°
2	NEU_HKA_0° NEU_FMA_0° VAL_TMA_3°
3	VAL_HKA_3° VAL_FMA_3° VAL_TMA_6°
4	NEU_HKA_0° VAL_FMA_3° NEU_TMA_0°
5	VAR_HKA_3° NEU_FMA_0° VAR_TMA_3°
6	VAR_HKA_9° NEU_FMA_0° VAR_TMA_6°
7	VAR_HKA_12° VAR_FMA_6° VAR_TMA_3°
8	VAR_HKA_3° VAR_FMA_3° VAL_TMA_3°
9	VAR_HKA_9° NEU_FMA_0° NEU_TMA_0°
10	VAR_HKA_12° NEU_FMA_0° VAR_TMA_3°

**Table 2:** Balancing results.

Patient number	Initial kinematic alignment					Initial mechanical alignment				
	Kinematic alignment	Femur preserving	Femur preserving lateral laxity	Tibia preserving	Tibia preserving lateral laxity	Mechanical alignment	Femur preserving	Femur preserving lateral laxity	Tibia preserving	Tibia preserving lateral laxity
1	**X**	**X**	**X**	**X**	**O**	**X**	**X**	**X**	**X**	**X**
2	**X**	**X**	**O**	**X**	**O**	**X**	**O**	**O**	**O**	**O**
3	**X**	**X**	**X**	**X**	**X**	**X**	**X**	**X**	**X**	**X**
4	**X**	**X**	**X**	**O**	**O**	**X**	**X**	**X**	**O**	**O**
5	**X**	**X**	**O**	**O**	**O**	**X**	**X**	**X**	**O**	**O**
6	**X**	**X**	**X**	**O**	**O**	**X**	**X**	**X**	**O**	**O**
7	**X**	**X**	**X**	**O**	**O**	**X**	**X**	**X**	**O**	**O**
8	**X**	**X**	**X**	**X**	**X**	**X**	**X**	**X**	**X**	**X**
9	**X**	**O**	**O**	**O**	**O**	**X**	**O**	**O**	**O**	**O**
10	**X**	**X**	**X**	**O**	**O**	**X**	**X**	**X**	**X**	**O**

### Preservation of the femoral component's alignment

Starting from MA, two TKAs could be functionally aligned, irrespective of the 90° lateral flexion laxity (20%).

Starting from KA, one TKA could be functionally aligned using the femur preserving technique, another two TKAs when lateral flexion laxity was allowed (30%). Adding 90° lateral flexion laxity allowed 20% more TKAs to be functionally aligned.

### Preservation of the tibial component's alignment

Starting from MA, six TKAs could be functionally aligned, adding one more if lateral flexion laxity was allowed (70%). Adding 90° lateral flexion laxity up to 6 mm allowed 10% more TKAs to be functionally aligned.

The difference in frequency of achieving FA when the alignment of the femoral or tibial component was preserved was significant (*p* = 0.025).

Starting from KA, six TKAs could be functionally aligned by preserving the tibial component's alignment, and another two TKAs if lateral flexion laxity was accepted (80%).

Two knees, VAL_HKA_3° VAL_FMA_3° VAL_TMA_6° and VAR_HKA_3° VAR_FMA_3° VAL_TMA_3° (20%) (Table 1), could not be functionally aligned without altering both components' position, regardless of the initial alignment and component preserving strategy, thus requiring a hybrid adjustment. One knee could only be functionally aligned starting from KA, preserving the tibial component's position and accepting lateral flexion laxity. On the other hand, one TKA could be functionally aligned using eight different combinations.

## DISCUSSION

The most important finding of the present study is that significantly more knees could be functionally aligned by preserving the tibial alignment and altering only the femoral alignment. However, 20% of knees could not be functionally aligned without adjusting both component alignments, even if a wider lateral flexion gap was accepted, thus requiring a hybrid adjustment.

FA is described as a strategy that can be delivered using robotics, combining patient bony anatomy and laxity soft tissue envelope to achieve a balanced knee [19]. Although the starting position and the boundaries are defined, FA fails to define what a balanced knee is—symmetrical or asymmetrical flexion gaps? More importantly, the femoral and tibial components can be moved independently, with each motion influencing one or more gaps. It is, therefore, crucial to define which component's position is preserved, the femoral or the tibial. The present study demonstrates that it is easier to functionally align the TKA by preserving the tibial component's position, which is to be expected since the tibial component influences flexion and extension more directly than the femoral component [25]. Accepting an increased lateral laxity at 90° flexion allowed one or two additional knees to be functionally aligned when starting from an MA or KA position, respectively. While 20% of TKAs required a hybrid adjustment, several TKAs could be functionally aligned with up to eight different strategies.

As the body of research for more personalized alignment strategies grows, it is crucial to define the patient's bony alignment and laxity and what strategy is being performed in order to better understand which strategy works for which patient. Whereas there are already two classifications of patient knee anatomy, the FKP [15] and the coronal plane alignment of the knee (CPAK) [18], the personalized alignment strategies remain ill‐defined. Even the fairly straightforward strategy of caliper‐verified KA is not always performed in the studies, although it is reported as such [4]. As the present study demonstrates, there are at least nine possible combinations to perform FA: femur preserving with or without lateral flexion laxity, tibial preserving with or without lateral flexion laxity, starting with KA and MA, as well as a hybrid option. A more detailed description of the applied functional alignment strategy will allow for a better understanding of which phenotype yields better outcomes with which alignment strategy [2].

The study demonstrates that starting of KA and preserving tibial alignment allows a more straightforward way to achieve FA. In addition, it allows a restoration of the knees in most knee phenotypes. This FA strategy, labelled iKA [26], has promising early results.

The pertinent question, what is FA, has not been answered, and FA is ill‐defined in many studies reported. Only when a clear nomenclature and reporting are put in place will reporting on outcomes using FA in TKA make results comparable. One then avoids an apples‐to‐oranges comparison.

Starting from MA and preserving the tibial component position results in a different final position of components compared to preserving the femoral component position. As the present study demonstrates, a hybrid solution, moving both components, might be required in certain instances. It is, however, crucial to report the starting position (MA vs. KA), the philosophy during balancing (which component position is preserved or aimed to be preserved), and how the knee is being balanced (symmetric vs. asymmetric flexion laxity).

We suggest the following classification for labelling FA in more detail:

Starting position (MA or KA)‐component position preservation (T or F)‐gap target (symmetric‐S or lateral flexion laxity‐LFL). iKA, for example, would be KA‐T‐LFL. If the coronal plane angles are restricted to 3°, an ‘r’ is to be added before the first abbreviation, rKA, for instance [27].

Some limitations need to be noted. The cohort size is small; however, due to the possible combinations and complexity of the analysis, this was left to the minimum, as per power analysis, to be able to demonstrate the topic in a digestible fashion. Therefore, not all possible phenotypes could be covered, but in this small and heterogeneous cohort, significant differences have been shown, and 20% of patients required a hybrid solution, demonstrating the complexity of the issue. Outcomes were not reported; however, the primary purpose of the study was to illustrate the complexity of the possible permutations of alignment strategies, not to compare outcomes. Due to the inability of any robotic system on the market to allow a post hoc balancing analysis of a real case, a macro was created to calculate the gaps. A rounding error might alter the results slightly, but each case was specifically verified for the 1 mm = 1° rule during balancing. All of the assumptions of balancing are based on similar ligament distractibility [10], which was the case in this study as well.

## CONCLUSION

In most TKAs, FA can be achieved from the MA and KA starting positions. Preserving the tibial alignment (iKA) allows FA more frequently than when the femoral alignment is preserved (KA). When the alignment of only one component is adjusted, even when accepting an increased 90° lateral flexion laxity, not all TKAs can be functionally aligned. Knees without a neutral phenotype alignment might require a hybrid strategy, where both components are adjusted. To better understand the different combinations behind FA, a more detailed nomenclature is required.

## AUTHOR CONTRIBUTIONS

Antonio Klasan and Michael T. Hirschmann conceived the study. All three authors did the measurements. Antonio Klasan did the analysis. Antonio Klasan and Michael T. Hirschmann wrote the first draft, and the coauthors revised it. All authors have read and approved the manuscript.

## CONFLICTS OF INTEREST STATEMENT

Antonio Klasan is an associate editor for the *Journal of Knee Surgery* and an editorial board member of *Archives of Orthopaedic and Trauma Surgery* and *Knee Surgery, Sports Traumatology, Arthroscopy*. Dragan Jeremic is a consultant for Medacta. A.J.N. is a consultant for Medacta, Think Surgical, Microport and Smith and Nephew. Thomas Neri declares no conflicts of interest. Thomas Jan Heyse has been paid for presentations by Smith and Nephew. Michael T. Hirschmann is a consultant for DePuy Synthes and Symbios.

## ETHICS STATEMENT

The study was performed under approval of a prospective data collection ethics board (AUVA Ethics Board 17/2021). All patients provided written consent to participate.

## Data Availability

Data are available upon request.
